# A double-blind, randomized, placebo-controlled study assessing the efficacy and tolerability of desvenlafaxine 10 and 50 mg/day in adult outpatients with major depressive disorder

**DOI:** 10.1186/1471-244X-13-94

**Published:** 2013-03-22

**Authors:** Michael R Liebowitz, Karen A Tourian, Eunhee Hwang, Linda Mele

**Affiliations:** 1 Columbia University, 134 East 93rd Street, Suite 201B, New York, NY, 10128, USA; 2 Pfizer Inc, 500 Arcola Road, Collegeville, PA, 19426, USA

**Keywords:** Depression, Adult, Antidepressive agents, Efficacy, Treatment, Drug safety

## Abstract

**Background:**

In an effort to establish the lowest effective dose of desvenlafaxine (administered as desvenlafaxine succinate), we assessed the efficacy, safety, and tolerability of 10- and 50-mg/day desvenlafaxine vs placebo for the treatment of major depressive disorder.

**Methods:**

Adult outpatients with DSM-IV–defined major depressive disorder and a 17-item Hamilton Rating Scale for Depression (HAM-D_17_) total score ≥20 were randomly assigned to receive placebo or desvenlafaxine (10 or 50 mg/day) after a 6- to 14-day single-blind placebo lead-in period in an 8-week, phase 3, fixed-dose trial. The primary efficacy measure was change from baseline in the HAM-D_17_ score analyzed using analysis of covariance. Efficacy analyses were conducted with the intent-to-treat population, using the last observation carried forward.

**Results:**

The intent-to-treat population included 673 patients. Change from baseline to final evaluation in adjusted HAM-D_17_ total scores was not significantly different comparing desvenlafaxine 10 mg/day (-9.28) and desvenlafaxine 50 mg/day (-8.92) with placebo (-8.42). There were no differences among treatment groups in the rates of treatment response or remission. Discontinuations due to adverse events occurred in 1.8%, 0.9%, and 1.8% of patients in the placebo and desvenlafaxine 10- and 50-mg/day groups, respectively. Overall rates of treatment-emergent adverse events with both doses were similar to placebo.

**Conclusions:**

Both doses of desvenlafaxine failed to separate from placebo. However, in a companion study reported separately, desvenlafaxine 50 mg, but not 25 mg, separated from placebo. Taken together, these studies suggest that 50 mg is the minimum effective dose of desvenlafaxine for the treatment of major depressive disorder.

**ClinicalTrials.gov Identifier:**

NCT00863798 http://clinicaltrials.gov/ct2/show/NCT00863798?term=00863798&rank=1.

## Background

Major depressive disorder (MDD) is a chronic, recurrent, and potentially disabling illness [1]. In the United States, the estimated lifetime prevalence of MDD is 17% [2] and the lifetime risk of developing MDD is estimated to be 10% to 25% in women and 5% to 12% in men [3]. There is a continuing need for antidepressant agents that are effective, safe, and well tolerated for both acute and long-term management of MDD.

Desvenlafaxine (administered as desvenlafaxine succinate) was approved by the United States Food and Drug Administration in 2008 for the treatment of adult patients with MDD [4] and is in the serotonin-norepinephrine reuptake inhibitor (SNRI) class of antidepressants. The efficacy and safety of desvenlafaxine at doses of 50, 100, 200, and 400 mg/day for the treatment of MDD has been evaluated in short-term, randomized, double-blind, placebo-controlled trials in adult outpatients with MDD [5-12]. Two pivotal trials demonstrated the efficacy of desvenlafaxine 50 mg/day (recommended dose) for the treatment of MDD in adult outpatients at 25 centers in the United States and 44 centers in Europe and South Africa [8,9]. Across all clinical studies of desvenlafaxine, doses above 50 mg/day were effective, but conferred no additional benefit; higher doses were associated with increased rates of adverse events (AEs). Treatment with desvenlafaxine was generally well tolerated in these studies.

The purpose of the current study was to evaluate the clinical effectiveness of lower dose desvenlafaxine (10 mg/day) and the recommended dose (50 mg/day) compared with placebo in outpatients with MDD. The study was designed to compare each desvenlafaxine dose group with placebo separately, with no comparisons between the desvenlafaxine 10- and 50-mg/day doses. The 17-item Hamilton Rating Scale for Depression (HAM-D_17_) total scores over 8 weeks, change from baseline in observer-rated depression scales and self-reported quality of life outcomes, and the safety/tolerability of desvenlafaxine were assessed. A companion study of low dose desvenlafaxine (25 and 50 mg/day) vs placebo was also recently performed and will be published [13].

## Methods

### Study design

This was a phase 3, double-blind, randomized, placebo-controlled, parallel-group study conducted in adult outpatients with MDD at 25 sites including private and institutional practice and research centers, within the United States between April 2009 and March 2010 (registered with ClinicalTrials.gov prior to first-subject first-visit, study identifier NCT00863798). The protocol received institutional review board or independent ethics committee approval before the study began, and the study was conducted according to the principles in the Declaration of Helsinki. All participants provided written, informed consent prior to study enrollment. The study included a 6- to 14-day single-blind placebo lead-in period and 8 weeks of double-blind treatment with desvenlafaxine 10 mg/day, desvenlafaxine 50 mg/day, or placebo. Patients returned for follow-up visits approximately 7 and 14 days after the last dose of study medication (for safety assessments only); there was no taper phase.

### Study population

#### Inclusion criteria

Eligible participants were medically healthy adult outpatients aged 18 years or older with a primary diagnosis of MDD based on criteria from the *Diagnostic and Statistical Manual of Mental Disorders*, 4th edition (text revision) confirmed through an investigator’s psychiatric clinical interview. Patients must also have had depressive symptoms ≥30 days before screening, total score ≥20 on the HAM-D_17_ at screening and baseline visit, score ≥2 on item 1 (depressed mood) of HAM-D_17_ at screening and baseline, and score ≥4 on the Clinical Global Impressions–Severity scale (CGI-S) at screening and baseline.

#### Exclusion criteria

Patients were excluded if they had received treatment with desvenlafaxine at any time in the past; known hypersensitivity to venlafaxine; significant risk of suicide; score of >3 on HAM-D_17_ item 3 (suicide) at screening or baseline; significant placebo response (≥25% decrease from screening in HAM-D_17_ total score at baseline); history of seizure disorder, gastrointestinal disease, or neoplastic disorder; or major acute illness within 90 days prior to screening. Patients with current psychoactive substance abuse or dependence (including alcohol), manic episode, posttraumatic stress disorder, obsessive-compulsive disorder, or a lifetime diagnosis of bipolar or psychotic disorder; current generalized anxiety disorder, panic disorder, or social anxiety disorder considered by the investigator to be primary (causing a higher degree of distress or impairment than MDD); or a clinically important personality disorder were also excluded, as were patients with any unstable hepatic, renal, pulmonary, cardiovascular (including uncontrolled hypertension or myocardial infarction within 180 days of the screening visit), ophthalmologic, neurologic, or other medical condition. Prohibited concomitant treatments included electroconvulsive therapy or formal psychotherapy (exclusive of supportive therapy) within 180 days of study day 1; venlafaxine within 90 days; investigational drugs or procedures, antipsychotics, or fluoxetine with 30 days; other antidepressants, monoamine oxidase inhibitors, anxiolytics, sumatriptan, naratriptan, zolmitriptan, and drugs with a similar mechanism of action, or tryptophan supplements within 14 days; and sedative hypnotics (other than zolpidem or zaleplon, allowed only during the first 14 days after randomization), herbal products intended to treat anxiety, insomnia, and depression, other psychotropic drugs or substances, or initiation of treatment with nonpsychopharmacologic drugs with psychotropic effects within 7 days of study day 1. Nonpsychopharmacologic drugs with psychotropic effects were permitted if the patient has been receiving a stable dose of the drug for at least 90 days before study day 1 and is expected to continue taking the drug without dose changes throughout the study.

### Treatment protocol

During the placebo lead-in period, patients received single-blind placebo for up to 14 days corresponding to the time in screening of 10±4 days. Following the placebo lead-in period, patients were randomly assigned to receive 8 weeks of double-blind treatment with placebo, desvenlafaxine 10 mg/day, or desvenlafaxine 50 mg/day with no titration period or taper phase. A 1:1:1 randomization schedule was generated by the Clinical Biostatistics Section of Pfizer Inc, (formerly Wyeth Research). Study site personnel called an automated system to receive subject randomization number and package number.

### Efficacy and safety assessments

The primary efficacy end point was change from baseline in HAM-D_17_ total score (observer-rated) at the final on-therapy (FOT) evaluation. HAM-D_17_ total scores were assessed at screening, baseline, and study days 7, 14, 21, 28, 42, and 56 (or upon early withdrawal from the study). The key secondary efficacy end point was the score at FOT on the Clinical Global Impressions–Improvement (CGI-I) [14]. Other efficacy variables included change from baseline in CGI-S, Montgomery-Ǻsberg Depression Rating Scale (MADRS) total score [15], and 6-item Hamilton Rating Scale for Depression (HAM-D_6_) score, as well as HAM-D_17_, MADRS, and CGI-I response rates and HAM-D_17_ remission rates. Scores were determined at the following time points: MADRS at baseline, and study days 14, 28, and 56 (or early withdrawal); CGI-S at screening, baseline, and study days 7, 14, 21, 28, 42, and 56 (or early withdrawal); CGI-I scores at study days 7, 14, 21, 28, 42, and 56 (or early withdrawal). Response on the HAM-D_17_, and MADRS were defined as ≥50% decrease in the respective total score from baseline; CGI-I response was defined as a score ≤2; HAM-D_17_ remission was defined as total score ≤7. Patient-rated secondary efficacy outcome measures included mean change from baseline in total scores on the World Health Organization 5-item Well-Being Index (WHO-5) and Sheehan Disability Scale (SDS; functional outcomes measures including work, social life, family life) SDS and WHO-5 assessments were obtained at baseline and study days 14, 28, and 56 (or early withdrawal). No efficacy assessments were collected at the follow-up visit. Safety assessments included monitoring of treatment-emergent adverse events (TEAEs), discontinuations due to AEs, and serious AEs (SAEs). AEs were evaluated at screening, baseline, study days 7, 14, 21, 28, 42, and 56, and at the 2 follow-up visits (study days 63 and 70).

### Statistical analysis

The biostatistics section of Pfizer (formerly Wyeth) carried out statistical analysis. It was estimated that a sample size of 216 per group would be needed with 2-sided ά=0.05 to provide 90% power to detect a difference between desvenlafaxine and placebo of 2.5 units of change (standard deviation of 8.0 units) on the HAM-D_17_ from baseline to FOT. To attain this sample size, the enrollment target was approximately 226 people randomized to each group.

The primary efficacy end point (change from baseline in HAM-D_17_ total score at the FOT evaluation for desvenlafaxine 10 mg/day or 50 mg/day vs placebo) was analyzed using analysis of covariance (ANCOVA) model with treatment as factor and baseline HAM-D_17_ total score as covariate for the intent-to-treat (ITT) population. Comparisons were made between each desvenlafaxine dose and placebo; the study was neither designed nor powered to compare the desvenlafaxine 10- and 50-mg/day dose arms. The ITT population included all randomly assigned patients who had a baseline primary efficacy evaluation, had taken at least 1 dose of double-blind study medication, and had at least 1 primary evaluation after the first dose of double-blind study medication. The key secondary end point (CGI-I score at the final on-therapy evaluation), was analyzed as a categorical variable using the Cochran-Mantel-Haenszel row-mean-score-difference test using ridit scores. Continuous secondary efficacy end points (CGI-S, MADRS total scores, HAM-D_6_ total scores, SDS total scores and subcomponents, and WHO-5 total scores) at the FOT evaluation were analyzed using ANCOVA with treatment as factor and corresponding baseline value as covariate. Response and remission rates for HAM-D_17_ and MADRS were analyzed with a logistic regression model with treatment as factor and baseline score as covariate. CGI-I response rate was analyzed using logistic regression with treatment as factor. All analyses were based on the ITT population with last observation carried forward (LOCF)-imputed data for missing assessments. Statistical testing was done 2-sided at the α=0.05 level. For the primary efficacy end point, the Hochberg step-up procedure was used to control for multiplicity associated with multiple active treatments. Testing of the key secondary end point occurred only when both active doses were superior to placebo on the primary end point to control the study-wise type I error rate across the primary and the key secondary end point of CGI-I, as well as across the 2 active dose arms. In this case, multiplicity arising from testing the key secondary end point in both doses would be controlled by a Hochberg step-up procedure. For other secondary efficacy end points, pairwise p-values will be reported without multiplicity control. For the safety population, summary tables and listings were generated for AEs, TEAEs, and SAEs. The safety population included all randomly assigned patients who had taken at least 1 dose of double-blind study medication.

## Results

### Patients

**Figure 1:**
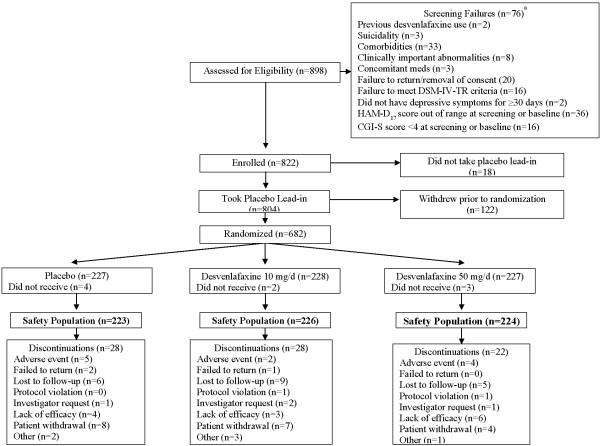
Population study flow.

A total of 898 patients were screened, 76 did not meet entry criteria and 18 were enrolled but did not take placebo lead-in (Figure 1). Of 804 patients who took the placebo lead-in, 122 withdrew prior to randomization, while 682 were randomized to treatment: 227 to placebo, 228 to desvenlafaxine 10 mg/day, and 227 to desvenlafaxine 50 mg/day. Of the 682 randomized patients, 9 did not receive treatment, leaving a sample of 673 patients who received at least 1 dose of study medication (safety population). The ITT population, consisting of patients who received at least one dose of study medication and had one post-baseline assessment, also included 673 subjects. Demographic and baseline clinical characteristics were similar among the desvenlafaxine 10 mg/day, desvenlafaxine 50 mg/day, and placebo treatment groups (Table 1). Seventy-eight (11.6%) patients discontinued from treatment prior to the end of the study (placebo group, n=28/223 [12.6%]; desvenlafaxine 10 mg/day, n=28/226 [12.4%]; desvenlafaxine 50 mg/day, n=22/224 [9.8%]). The most common reasons for early discontinuation were withdrawal by patient, lost to follow-up, and lack of efficacy.

**Table 1:** Demographic and baseline characteristics of safety population by treatment group

**Characteristic**	**Placebo**	**Desvenlafaxine**	**Desvenlafaxine**
	**(n = 223)**	**10 mg/d (n = 226)**	**50 mg/d (n = 224)**
**Age, y**	42 ± 13	41 ± 14	43 ± 14
**Age group, n (%)**			
18-64 y	215 (96)	212 (94)	213 (95)
>64 y	8 (4)	14 (6)	11 (5)
**Female, n (%)**	139 (62)	135 (60)	135 (60)
**Race, n (%)**			
White	176 (79)	199 (88)	177 (79)
Black	38 (17)	20 (9)	37 (17)
Asian	4 (2)	3 (1)	2 (1)
Other	5 (2)	4 (2)	8 (4)
**Weight, kg**	87 ± 24	88 ± 27	91 ± 26
**Body mass index, kg/m**^**2**^	30 ± 7	31 ± 9	32 ± 8
**Baseline HAM-D**_**17 **_**score**	23 ± 3	23 ± 2	23 ± 3
**Duration of current depressive episode, mo**	34 ± 81	26 ± 48	31 ± 76

Data are presented as mean ± standard deviation unless otherwise indicated.

d, day; HAM-D_17_, 17-item Hamilton Rating Scale for Depression; ITT, intent-to-treat; kg, kilogram; m, meter; mo, months; y, years.

### Efficacy evaluation

**Figure 2:**
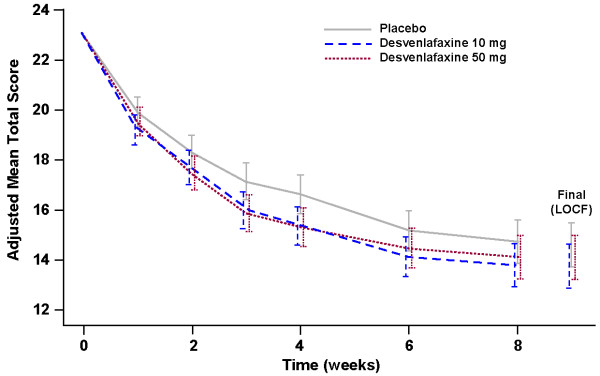
**Adjusted mean scores on HAM-D**_**17 **_**(ANCOVA), LOCF data, ITT population.**

Figure 2 shows the adjusted mean scores on the HAM-D_17_ from randomization to week 8 (and LOCF). The primary efficacy end point of the adjusted mean change from baseline in the HAM-D_17_ total score at the FOT evaluation did not reach statistical significance for patients in the desvenlafaxine 10 mg/day (-9.28) or desvenlafaxine 50 mg/day (-8.92) treatment groups vs placebo (-8.42; *P*=0.175 and *P*=0.421, respectively). The adjusted mean difference (95% confidence interval [CI]) vs placebo was 0.86 (-0.38, 2.10) and 0.51 (-0.73, 1.75) for the desvenlafaxine 10 mg/day and 50 mg/day treatment groups, respectively.

**Figure 3:**
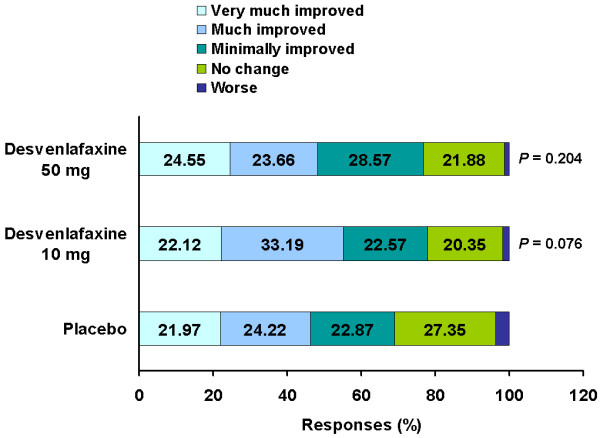
CGI-I at final on-therapy visit (LOCF), ITT population.

For the key secondary efficacy end point of CGI-I score at FOT, percentages of patients who were rated as “very much improved” or “much improved” were 55.3% and 48.2% in the desvenlafaxine 10 mg/day and 50 mg/day treatment groups, respectively, compared with 46.2% in the placebo group; no significant differences were observed among the desvenlafaxine treatment groups vs placebo before and after the multiplicity control (Figure 3). Similarly, the change from baseline in CGI-S, MADRS total score, and HAM-D_6_ total score did not differ between the desvenlafaxine 10 mg/day and 50 mg/day treatment groups vs placebo (Table 2). In addition, *P*-values were above 0.05 when the desvenlafaxine 10 mg/day or 50 mg/day treatment groups were compared with placebo for rates of HAM-D_17_ response (44%, 41%, and 38%, respectively); HAM-D_17_ remission (23% and 17% vs 19%, respectively); MADRS response (42% and 41% vs 38%, respectively); or CGI-I response (55% and 48% vs 46%, respectively). However, pairwise p-values vs. placebo were below 0.05 for the adjusted mean change from baseline in SDS Work Studies component scores for both desvenlafaxine 10 mg/day and 50 mg/day vs placebo (change of -1.10, -1.14, and -0.61, respectively; Table 2). p-values for the adjusted mean change from baseline in WHO-5 total score, and SDS total score and social life components were also below 0.05 for desvenlafaxine 10 mg/day vs placebo.

**Table 2:** Adjusted mean change from baseline in total scores on various secondary efficacy outcome measures (LOCF), ITT Population

	**Adjusted mean (SE) change from baseline**		
**Secondary outcome**	**Placebo**	**Desvenlafaxine 10 mg/d**	**Desvenlafaxine 50 mg/d**
MADRS total score	–9.87 (0.63)	–11.28 (0.63)	–10.76 (0.63)
CGI-S total score	–1.08 (0.07)	–1.23 (0.07)	–1.11 (0.07)
HAM-D_6_ total score	–4.75 (0.27)	–5.49 (0.27)	–5.19 (0.27)
SDS total score	–2.63 (0.48)	–4.09 (0.48)^†^	–3.78 (0.49)
SDS Work Studies component	–0.61 (0.17)	–1.10 (0.17)^†^	–1.14 (0.17)^†^
SDS Social Life component	–1.08 (0.18)	–1.58 (0.17) ^†^	–1.36 (0.18)
SDS Life/Home Responsibilities	–0.98 (0.17)	–1.43 (0.17)	–1.20 (0.17)
WHO-5 total score	2.96 (0.36)	4.51 (0.35)^‡^	3.73 (0.36)

^†^p<0.05 and ^‡^p<0.01; ANCOVA model using treatment as factor and baseline as covariate.

CGI-S, Clinical Global Impressions–Severity; HAM-D_6_, 6-item Hamilton Rating Scale for Depression; ITT, intent to treat; LOCF, last observation carried forward; MADRS, Montgomery-Åsberg Rating Scale for Depression; SDS, Sheehan Disability Scale; WHO-5, World Health Organization 5-item Well-being Index.

### Safety evaluation

Treatment-emergent adverse events were reported by 147 patients (66%) in the placebo group, 155 patients (69%) in the desvenlafaxine 10 mg group, and 154 patients (69%) in the desvenlafaxine 50 mg/day group. The most common TEAEs (incidence ≥5% in any treatment group) during the on-therapy period are shown in Table 3. Adverse events resulted in discontinuation of treatment for 10 of 673 patients (1.5%) overall (4 patients [1.8%] in the placebo group; 2 patients [0.9%] in the desvenlafaxine 10 mg/day treatment group; and 4 patients [1.8%] in the desvenlafaxine 50 mg/day treatment group) during the on-therapy period. Serious adverse events were reported by 5 patients (0.7%). Of these 5 patients, 4 had an SAE during the on-therapy period (placebo, n=2 [noncardiac chest pain and appendicitis in 1 patient; scrotal abscess in 1 patient]; desvenlafaxine 10 mg/day, n=1 [urinary retention]; and desvenlafaxine 50 mg/day, n=1 [pulmonary embolism]). The pulmonary embolism in this 53-year-old female was determined by the study investigator to be related to study medication. She had a nonproductive cough that began between screening and randomization which did not respond to a 10-day course of antibiotics. On study days 8 to 11 she reported edema in the ankles. A CT scan on day 19 showed pulmonary embolism in the right lung. She received treatment for the pulmonary embolism and discontinued from the study on day 20.

**Table 3:** Number (%) of patients reporting TEAES with incidence ≥5% in any group, on-therapy period, safety population

	**Placebo**	**Desvenlafaxine**	**Desvenlafaxine**
	**(n=223)**	**10 mg/d (n=226)**	**50 mg/d (n=224)**
**Any TEAE**	147 (66)	155 (69)	154 (69)
**Gastrointestinal disorders**			
Constipation	5 (2)	4 (2)	15 (7)
Diarrhea	11 (5)	14 (6)	17 (8)
Dry mouth	11 (5)	17 (8)	20 (9)
Nausea	14 (6)	21 (9)	32 (14)
**General disorders**			
Fatigue	4 (2)	7 (3)	13 (6)
**Infections**			
Upper RTI	11 (5)	18 (8)	12 (5)
**Metabolism and nutrition disorders**			
Decreased appetite	8 (4)	9 (4)	20 (9)
**Nervous system disorders**			
Dizziness	12 (5)	9 (4)	16 (7)
Headache	17 (8)	18 (8)	16 (7)
**Psychiatric disorders**			
Insomnia	8 (4)	11 (5)	15 (7)

RTI, respiratory tract infection; TEAE, treatment-emergent adverse event.

Mean changes in vital signs from baseline to the FOT assessment were not significantly different comparing desvenlafaxine 10 mg/day or 50 mg/day vs placebo for supine pulse rate, supine systolic pressure, and supine diastolic pressure. Mean decreases in weight in the desvenlafaxine 50 mg/day group were significant compared with baseline and with mean changes in the placebo group at weeks 1, 2, 3, 4, 6, 8, and FOT, whereas the desvenlafaxine 10 mg/day group was not significantly different from baseline or placebo at any time point. At FOT, adjusted mean (SE) changes in weight from baseline were 0.11 (0.16) kg, -0.03 (0.16) kg, and -0.55 (0.16) kg for the placebo, 10 mg/day, and 50 mg/day groups, respectively. No participant on placebo, 2 in the 10 mg/day group (<1%), and 4 in the 50 mg/day group (1.8%) gained ≥7% of their baseline body weight during treatment, while 2 (<1%), 5 (2.2%) and 3 (1.3%) lost ≥7% of their baseline body weight, respectively.

## Discussion

Desvenlafaxine is an SNRI that has demonstrated efficacy at doses of 50 to 400 mg/day. The objective of the current study was to evaluate the efficacy and tolerability of a lower dose of desvenlafaxine (10 mg/day) compared with placebo in adult outpatients with MDD. Treatment with desvenlafaxine 10 mg/day did not separate from placebo on the primary efficacy measure, HAM-D_17_ improvement from baseline, nor on observer-rated secondary depression measures. However, patient-related secondary measures showed improvements from baseline in scores on the SDS and WHO-5 for patients who received desvenlafaxine 10 mg/day compared with those who received placebo. Safety findings for desvenlafaxine 10 and 50 mg/day were similar to those previously reported for desvenlafaxine treatment [8,9] and were consistent with known safety findings of other antidepressant medications.

Although the efficacy of desvenlafaxine 50 mg has been demonstrated in previous MDD trials [8,9] in the current study, this dose did not reach statistical significance compared with placebo for the primary efficacy measure. However, it should be noted that approximately 50% of antidepressant trials of effective drugs fail to meet clinical superiority vs placebo [16]. A high placebo response (>30% symptom reduction on the HAM-D score) [16] and baseline HAM-D score <25 [17] also may reduce the likelihood of reaching statistical differences between antidepressant treatment and placebo. In the current study, the placebo lead-in period used to exclude placebo responders did reduce the magnitude of mean change in HAM-D_17_ scores from the approximately 40% to 45% reductions observed for placebo in previous MDD trials [8,9]. Nonetheless, participants in the placebo group in this study had a mean HAM-D_17_ symptom reduction of 36% over 8 weeks, and both the placebo and desvenlafaxine 50 mg/day groups had baseline HAM-D_17_ scores of ~23.

The strengths of this study include the randomized, placebo-controlled trial design and the broad range of efficacy and safety measures employed. A wide range of efficacy measures were studied utilizing both observer-rated (HAM-D_17_, CGI-I, MADRS) and patient-rated (WHO-5, SDS) assessment measures. In the current study, there were no statistical improvements seen with either dose of desvenlafaxine in the observer-rated measures, but some of the self-rated measures did show statistical differences vs placebo. Several features of observer- and self-assessment tools (e.g., HAM-D_17_ vs SDS) are important to consider when interpreting results [18-21]. Observer-rated scales take advantage of a trained clinical researcher’s ability to assess the severity of a patient’s symptoms in a clinical context, whereas self-rating scales may be affected by factors such as the inherent reflection of the patient’s individual experience, willingness for self-disclosure, self-perception, language skills, and cognitive capabilities [18]. In addition, self-ratings may be more likely to take into account symptoms not classically considered MDD, such as pain and anxiety [21]. The self-rated scales in the current study focused on global well-being and disability rather than depressive symptoms, which may have contributed to the differences that emerged; however, the lack of concordance between the observer-rated and patient-rated scales in this study limits the conclusions that can be drawn about the efficacy of desvenlafaxine 10 mg/day.

A limitation of the current study was the fixed-dose design, which did not provide the ability to up-titrate desvenlafaxine in patients who may have needed or may have tolerated higher doses, and results may not be fully generalizable to clinical practice. Another limitation is that no comparisons can be made between the desvenlafaxine 10 and 50 mg/day doses, since this study was not designed nor powered to compare the treatment arms.

In the absence of the clinical efficacy of desvenlafaxine 50 mg/day, the results of this study with desvenlafaxine 10 mg/day may be considered inconclusive and the interpretation of the data is difficult. However, a companion study comparing low-dose desvenlafaxine (25 mg/day) and desvenlafaxine (50 mg/day) with placebo did support the clinical superiority of desvenlafaxine 50 mg/day over placebo, and found no significant improvement with 25 mg/day in either observer- or patient-rated measures [13]. The data from the companion study in concert with the findings obtained in the current study suggest that desvenlafaxine is not effective at doses lower than 50 mg/day.

## Conclusions

In this phase 3, multicenter, randomized, double-blind study, neither desvenlafaxine 10 mg/day nor desvenlafaxine 50 mg/day separated from placebo on the primary efficacy end points in patients with MDD. Treatment with desvenlafaxine 10 mg/day and 50 mg/day was generally safe and well-tolerated, with TEAEs observed in this study comparable to those observed in previously reported phase 3 studies of desvenlafaxine treatment for MDD. These results, in conjunction with those from a similar study [13], suggest that doses of desvenlafaxine below 50 mg/day are not effective for treatment of MDD.

## Abbreviations

AEs: Adverse events; ANCOVA: Analysis of covariance; CGI-I: Clinical Global Impressions–Improvement scale; CGI-S: Clinical Global Impressions–Severity of Illness scale; FOT: Final on-therapy; MDD: Major depressive disorder; HAM-D6: 6-item Hamilton Rating Scale for Depression; HAM-D17: 17-item Hamilton Rating Scale for Depression; ITT: Intent-to-treat; MADRS: Montgomery-Ǻsberg Depression Rating Scale; SDS: Sheehan Disability Scale; SAEs: Serious adverse events; SNRI: Serotonin-norepinephrine reuptake inhibitor; RTI: Respiratory tract infection; TEAEs: Treatment-emergent adverse events; WHO-5: World Health Organization 5-item Well-Being Index

## Conflict interest

Dr. Liebowitz has received reimbursement for the development of the clinical study report. He is Managing Director of The Medical Research Network LLC, which received contracts as a site for this clinical trial. Dr. Tourian, Dr. Hwang and Ms. Mele are full-time Pfizer employees.

## Authors’ contributions

Conception and design: Dr. Liebowitz, Dr. Tourian, Ms. Mele, Dr. Hwang, Acquisition of Data: Dr. Liebowitz, Dr. Tourian, Ms. Mele, Analysis and Interpretation of Data: Dr. Liebowitz, Dr. Tourian, Ms. Mele, Dr. Hwang, Drafting the manuscript: Dr. Liebowitz, Dr. Tourian, Ms. Mele, Dr. Hwang, Revisions for intellectual content: Dr. Liebowitz, Dr. Tourian, Ms. Mele, Dr. Hwang, Final Approval of the Completed Manuscript: Dr. Liebowitz, Dr. Tourian, Ms. Mele, Dr. Hwang. All authors read and approved the final manuscript.

## Pre-publication history

The pre-publication history for this paper can be accessed here:

http://www.biomedcentral.com/1471-244X/13/94/prepub
